# Disparities in Cardiovascular Disease and Type 2 Diabetes Risk Factors in Blacks and Whites: Dissecting Racial Paradox of Metabolic Syndrome

**DOI:** 10.3389/fendo.2017.00204

**Published:** 2017-08-31

**Authors:** Kwame Osei, Trudy Gaillard

**Affiliations:** ^1^Professor Emeritus of Medicine and Exercise Physiology, Division of Endocrinology, Diabetes and Metabolism, The Ohio State University, Columbus, OH, United States; ^2^College of Nursing, University of Cincinnati, Cincinnati, OH, United States

**Keywords:** Blacks, Whites, cardiovascular diseases, type 2 diabetes, lipids/lipoproteins, metabolic syndrome, inflammation

## Abstract

Cardiovascular diseases (CVD) remain as the leading cause of mortality in the western world and have become a major health threat for developing countries. There are several risk factors that account for the CVD and the associated mortality. These include genetics, type 2 diabetes (T2DM), obesity, physical inactivity, hypertension, and abnormal lipids and lipoproteins. The constellation of these risk factors has been termed *metabolic syndrome* (MetS). MetS varies among racial and ethnic populations. Thus, race and ethnicity account for some of the differences in the MetS and the associated CVD and T2DM. Furthermore, the relationships among traditional metabolic parameters and CVD differ, especially when comparing Black and White populations. In this regard, the greater CVD in Blacks than Whites have been partly attributed to other non-traditional CVD risk factors, such as subclinical inflammation (C-reactive protein), homocysteine, increased low-density lipoprotein oxidation, lipoprotein a, adiponectin, and plasminogen activator inhibitor-1, etc. Thus, to understand CVD and T2DM differences in Blacks and Whites with MetS, it is essential to explore the contributions of both traditional and non-traditional CVD and T2DM risk factors in Blacks of African ancestry and Whites of Europoid ancestry. Therefore, in this mini review, we propose that non-traditional risk factors should be integrated in defining MetS as a predictor of CVD and T2DM in Blacks in the African diaspora in future studies.

## Introduction

Metabolic syndrome (MetS) is a constellation of risk factors that predict future cardiovascular disease (CVD) and type 2 diabetes (T2DM) (1–6). MetS affects over 70–80 million Americans in the US and increases with age (2–4). For the past four decades, there has been tremendous debate on the significance of MetS *per se* when compared to the sum of individual components of MetS. These risk factors include obesity [waist circumference (WC)], blood pressure (BP), fasting triglycerides, high-density lipoprotein cholesterol (HDL-C), and fasting glucose level (1–6). Because of ethnic differences among the MetS components, the International Diabetes Federation (IDF), have provided different guidelines and criteria based on gender, race and ethnicity (7). In this regard, National Cholesterol Education Program-Adult Treatment Panel III (NCEP-ATP) criteria require three or more of the five components to constitute MetS (Table 1). The ATP III does not have race/ethnic based MetS criteria and requires no prerequisite (1, 3, 5, 8). In contrast, the IDF criteria require WC as a prerequisite in addition to two or more parameters for the diagnosis of MetS (Table 2). Thus, the prevalence of MetS and its components differ among different racial and ethnic populations (Table 3) (9–14). These metabolic risk factors account in part, for the ethnic differences in cardiovascular mortality and morbidity (9, 12–14). Therefore, this mini review will explore further the rationale for the potential disparities in MetS and its outcomes in Blacks of African ancestry and Whites of Europoid origin residing in diverse geographic locations.

**Table 1:** National cholesterol education program-adult treatment panel III definition of metabolic syndrome (8).

Components	Criteria
Waist circumference	Men >40 inches or >102 cm
	Women >35 inches or >88 cm
Fasting triglycerides	≥150 mg/dl
Blood pressure	SBP ≥ 130 mmHg and/or DBP ≥ 85 mmHg
Fasting glucose	>100 mg/dl
High-density cholesterol (HDL)	Men > 40 mg/dl
	Women > 50 mg/dl

**Table 2:** International diabetes federation metabolic syndrome worldwide definition (7).

Component	Criteria
Central obesity	Waist circumference
	Ethnicity specific (Refer to Table 3)
**Plus any two of the following**	
Raised triglycerides	≥1.7 mmol/l (150 mg/dl) or
	Specific treatment for this lipid abnormality
Reduced high-density cholesterol	<1.03 mmol/l (40 mg/dl) in males
	<1.29 mmol/l (50 mg/dl) in females or
	Specific treatment for this lipid abnormality
Raised blood pressure	Systolic: ≥130 mmHg or
	Diastolic: ≥85 mmHg or
	Treatment of previously diagnosed hypertension
Raised fasting plasma glucose	Fasting plasma glucose ≥ 5.6 mmol/l (100 mg/dl) or
	previously diagnosed type 2 diabetes.
	If > 5.6 mmol/l or 100 mg/dl, oral glucose tolerance test is strongly recommended but is not necessary to define presence of the syndrome.

**Table 3:** Supplement: country/ethnic-specific cutoff points for waist circumference (WC) as described by the international diabetes federation (7).

Country/ethnic group	WC (as measure of central obesity)
Europoids	Male ≥ 94 cm
	Female ≥ 80 cm
South Asians	Male ≥ 90 cm
	Female ≥ 80 cm
Chinese	Male ≥ 90 cm
	Female ≥ 80 cm
Japanese	Male ≥ 85 cm
	Female ≥ 80 cm
Ethnic South and Central Americans	Use South Asian recommendations until more specific data are available
Sub-Saharan Africans	Use European data until more specific data are available
Eastern Mediterranean and Middle East	Use European data until more specific data are available (Arab) populations

## Prevalence of MetS in Blacks and Whites

Several previous studies have shown that the prevalence of MetS is lower in Blacks when compared to Whites, irrespective of their geographic location (9–14). In fact, the National Health and Nutrition Examination Survey (NHANES III) reported that African-Americans have lower prevalent rates of MetS than Whites in the US (15–21). In particular, African-American males have lower MetS when compared with their White male counterparts (18–21). Recently, the NHANES and other authors have reported higher prevalence of MetS in African-American women (20, 22, 23). Furthermore, Afro-Caribbean’s living in the United Kingdom (9, 14) and Blacks in South Africa (24, 25) have lower prevalence of MetS than their White counterparts. Paradoxically, despite the lower prevalence of MetS, Blacks suffer disproportionately higher CVD and T2DM and their associated morbidity and mortality than their White counterparts (12, 15, 17–21, 26). Thus, these metabolic and CVD paradoxes suggest that the processes leading to CVD and T2DM appear to differ in Blacks and Whites.

## Disparities in Insulin Resistance (IR) in Blacks and Whites

The pathophysiology of MetS has been extensively studied for over four decades among racial/ethnic populations by several investigators. The well accepted pathophysiologic lesion underlying MetS is IR/hyperinsulinemia (10–12, 27–32). Whether the IR directly or *via* the hyperinsulinemia determine MetS remains debatable. Theoretically, insulin plays a critical role in hepatic lipid synthesis, renal sodium reabsorption, arterial tissue growth, and vascular reactivity. These physiologic actions of insulin can partly play a role in the development of clinical obesity, hypertension (HTN), and atherosclerosis by binding to insulin receptor substrate 1, that stimulates post receptor signaling pathways. Although, these insulin-mediated mechanisms are plausible, direct proof of these concepts remains uncertain in *in vivo* in humans. Furthermore, there is clear evidence that IR varies among different racial/ethnic populations (10–12, 27–33). In this respect, Blacks (youth and adults) manifest greater IR and insulin levels than their White counterparts (12, 13, 24, 29, 30). Factors that contribute to IR include genetics, obesity and lower physical activity, smoking which are higher in Blacks than Whites (27–32). Finally, the relationships of IR and the components of MetS vary among different racial/ethnic populations.

## Disparities in HTN in Blacks and Whites

Hypertension is one of the most common non-communicable diseases in the world and it is projected to affect over 1 billion people worldwide (17, 34). Thus, BP constitutes a major component of MetS. Consequently, HTN increases the prevalence of CVD outcomes, such as stroke, congestive heart failure, kidney failure and cognitive impairment/dementia in both developed and underdeveloped countries among racial and ethnic populations (17). In this regard, Blacks are more disproportionately affected by HTN and its comorbid conditions. In the US, the prevalence of HTN is 40–50% in African-Americans when compared to 25–30% in Whites (35, 36). Furthermore, the prevalence of HTN associated complications are higher in Blacks than Whites. Compared to Whites, Blacks have higher prevalence of heart failure, by twofold, stroke, by twofold to fourfold and kidney failure, by twofold to fourfold in the US (11–13, 34–36). These could partly explain the higher disparities in CVD outcomes in Blacks than Whites. Although, most patients have essential HTN, some authorities believe IR might be the underpinning lesion of MetS, especially regarding BP (10, 11, 34–36). However, the mechanism(s) by which IR causes higher BP remains debatable. Indeed, while IR correlates with BP in Whites, this is not the case in Blacks. The reasons for the IR and BP paradox in Blacks remain uncertain.

## Disparities in Glucose Tolerance and T2DM in Blacks and Whites

Diabetes has become a global epidemic, especially in minority populations. The prevalence of glucose intolerance, prediabetes and T2DM is higher in Blacks than Whites who reside in diverse geographic locations (5, 16, 17, 26, 30, 37, 38). There is a growing evidence for epidemic of T2DM in minority populations (39). In this context, it is estimated that the prevalence of T2DM is 1.5–2× higher in Blacks than Whites in the US (37). Furthermore, these racial differences in T2DM have been observed in Blacks residing in United Kingdom (6, 10, 14, 26, 40) and South Africa (6, 24, 25). The reasons for the racial disparity in diabetes in Blacks and Whites are unknown, but appear to be multifactorial including genetic inheritance and environment factors (physical inactivity, nutrition, obesity, lower socioeconomic status, smoking, etc.).

Pathogenically, glucose intolerance and T2DM have been attributed to alterations in beta cell function, decreased insulin secretion and IR (both peripheral and hepatic) (27–33). Blacks residing in the western world are more hyperinsulinemic and manifest greater IR than their White counterparts, independent of obesity (10, 11, 29, 31–33). The higher insulin responses to glucose and non-glucose stimulation in non-diabetic Blacks can be attributed to greater beta cell secretion and defective hepatic extraction or insulin clearance (31–33). Although, the molecular mechanism for the altered beta cell secretion and insulin kinetics and IR remain uncertain in patients with T2DM, there is a clear evidence for genetic (and epigenetic) as well as environmental factors such as physical inactivity and overweight/obesity which are higher in Blacks and Whites.

## Disparities in Lipids and Lipoproteins in Blacks and Whites

Alterations in lipids and lipoproteins that play pathogenic role in atherosclerosis are major components of MetS (1–3). Typically, the MetS is associated with higher serum triglycerides and lower HDL-C and higher small dense low-density lipoprotein (LDL) (apo B) particles (1, 41–48). This has been attributed in part to the underlying IR and obesity (1–5, 31–33). These abnormalities exist in patients with prediabetes and progresses with time (48). However, the relationships between IR and lipids and lipoproteins differ among racial and ethnic populations (49–55). In this regard, IR is associated with lower triglycerides and higher HDL-C in Blacks than Whites (29–33, 51, 52). Thus, these relationships are inverse and paradoxical in Blacks with MetS than Whites residing in diverse geographic locations. Therefore, it has been postulated that, compared to Whites, Blacks with and without MetS have favorable anti-atherogenic lipid and lipoprotein profiles that could theoretically protect them against CVD, but this is not the case. Therefore, fasting triglycerides and trig/HDL-C ratios appear not to be markers of IR in Blacks (29–33, 51, 52). The reasons for this metabolic paradox remain unknown. We (22, 31–33, 48) and others (27, 46, 47, 51) have attributed the lower triglycerides in Blacks than Whites to possible defective hepatic synthesis of very low-density lipoprotein (VLDL) as measured by particle concentrations, numbers and sizes derived from Nuclear Magnetic Resonance (NMR) spectroscopy (41–48). In this regard, the proposed underlying defect has been attributed in part to hepatic lipase gene mutation (45–47). In addition Mijkoviv-Gacic et al. (46) and Frazier-Wodds et al. (47) reported lower NMR-derived total, medium, and small VLDL particles in African-Americans and Afro-Caribbeans when compared to Whites. Hence, it can be inferred that the racial and ethnic differences in lipids and lipoprotein profile cannot explain the paradoxically higher and excess CVD mortality and morbidity in Blacks when compared to Whites (17–20). Answers to these questions deserve further elucidation. In support of this concept, the number of large HDL particles are greater in non-diabetic Blacks than Whites (46–48). Furthermore, using NMR-derived lipoprotein, LDL particle sizes and numbers are consistent with the well accepted pathogenic role in atherosclerosis in Whites, but not so for Blacks (46–48). In fact, Blacks have large LDL particles, which are more buoyant and less atherogenic than Whites. These findings are consistent with those from the Dallas Heart Study (45) and Multi-Ethnic Study of Atherosclerosis (44, 54).

## Disparities in Overweight/Obesity in Blacks and Whites

Obesity has become epidemic globally especially in minority populations (1, 5, 6, 15, 16, 24, 33, 38, 40). This is particularly so for Blacks of African ancestry residing in urban and inner cities of the western world when compared with their White counterparts (11, 24, 55–62) and Blacks residing in rural areas in Africa (6, 14, 40, 55–57, 59). In fact, the prevalence of overweight and obesity approaches 70% in Blacks when compared to 50% in Whites in the US (5, 20). The obesity with the associated IR accounts partly for the myriads of diseases such as CVD, T2DM, HTN, obstructive sleep disorder, CHF, stroke and cancer. In this context, obesity with the truncal obesity (WC) is regarded as a pivotal clinical indicator of MetS (5–7, 55, 56, 63, 64). However, the biological significance of WC varies and remains debatable among racial and ethnic populations (7, 55, 56, 63, 64). Specifically, the regional body fat distribution and composition tend to vary among ethnic and racial populations. For example, for identical body mass index, Blacks have lower intra-abdominal visceral adiposity (VAT) when compared to Whites (63, 64). Moreover, the corresponding subcutaneous fat depot (SAT) tends to be the same or greater in Blacks than Whites in both genders (63, 64). The reasons for the racial and ethnic disparities in VAT and SAT distribution remain uncertain and debatable.

The metabolic consequences of obesity/overweight have been attributed to the biological activity of the adipocytes and adipose tissues unique to the fat distribution such as visceral and subcutaneous adiposity or fat deposition. In particular, intra-abdominal VAT in general is more biologically active and it is associated with systemic and hepatic IR in several non-Black populations (57). However, despite the greater IR in Blacks, the corresponding VAT is lower in Blacks than Whites (63, 64). The reasons and the mechanisms of the VAT and IR paradox in Blacks remain unknown and debatable.

## Disparities in Lipid Oxidation and Subclinical Inflammation in Blacks and Whites

Recently, there has been increasing interest in the role of non-traditional markers in CVD and T2DM which also underpin MetS. In this context, lipid oxidation is an important etiologic factor in CVD and T2DM. In particular, oxidized LDL serves as the foundation or causative factor for vascular endothelial injury and the development of fatty streak or plaque and early atherogenesis (65, 66). The oxidative processes are mediated through changes in super-oxide dismutase or free oxygen radicals (67, 68). Clinically, these processes can be assessed as oxidized LDL and F_2_ Isoprostanes. This concept suggests that reducing LDL oxidation could also potentially reduce CVD and T2DM. In this regard, to minimize or reduce LDL oxidation, HDL has been implicated as a potent antioxidant agent especially against LDL particle oxidation (69–72). Furthermore, HDL is regarded as anti-inflammatory particle with anti-vascular injury properties (71, 72).

Previous studies have demonstrated that HDL is a potent anti-atherogenic particle. In addition, Apo AII, the largest HDL protein, has been postulated to play a major role as anti-atherogenic. In this context, both Apo AII and HDL-C levels are higher in Blacks than Whites with and without T2DM and MetS (41, 42, 52–54). But these higher HDL particles do not appear to protect against CVD in Blacks when compared to Whites (13–18). The reasons are unknown. Another potential mechanism for HDL oxidation is paraoxonase. In this context, we should note that, some of the anti-atherogenic biological properties of HDL have also been partly attributed to paraoxonase enzyme 1 (PON1), a major component of HDL (66–70). PON1 which cosegregates with HDL in the circulation is partly responsible for the antioxidant (oxidized LDL) and anti-inflammatory properties [C-reactive protein (CRP)] of HDL (66–69). In this regard, oxidized LDL level and CRP are 50% higher in Blacks when compared to Whites (69). It should be noted that subclinical inflammation is considered as an important etiologic factor for both CVD and T2DM. In this context, CRP levels which are indicators of subclinical inflammation are significantly elevated in Blacks than Whites who have increased propensity for CVD, T2DM and MetS (65–69, 73). These findings indicate that the higher HDL levels appear to be dysfunctional in Blacks compared to Whites (69). In support of the HDL dysfunctionality concept, Blacks have greater large HDL particles than Whites (44–46). Similar to the concept of HDL dysfunctionality in Blacks, Dodani et al. (70) demonstrated that HDL function is lower or impaired in South Asian Indians, a population with extreme propensity for CVD and its outcomes, when compared to Whites.

## Disparities in Adipocytokines and Other Non-Traditional Risk Factors in Blacks and Whites

Non-traditional CVD markers include aipocytokines such as adiponectin, interleukin 6 (IL-6), tumor necrosis factor alpha, homocysteine plasminogen activator inhibitor-1 (PAI-1), as well as platelate aggregration factor, etc., which are mostly derived from adipocytes (74–82). Physiologically, adiponectin, 244 aa protein which is solely derived from adipocytes, is a potent endogenous insulin sensitizer (peripheral and hepatic) and tends to increase with weight loss (physical exercise, bariatric surgery, etc.) and use of some drugs such as thiazolidinediones. However, the circulating levels of adiponectin vary according to race and ethnicity. In this regard, Blacks with and without T2DM, who are normal weight, overweight or obese, have lower adiponectin levels when compared to their White counterparts (74–77). Thus, the role of adiponectin in CVD and T2DM warrants further investigation. Whether the adiponectin synthesis is lower or its clearance is higher in Blacks compared to Whites remain to be elucidated.

There are other non-traditional CVD and Type 2 DM risk factors. We propose that these non-traditional risk factors play critical role in CVD and its outcomes in African-Americans than Whites (81, 82). These other non-traditional risk factors in the development of CVD include alterations in homocysteine, PAI-1, Lp (a) as well as platelet aggregation factors and hypercoagulability. However, whether normalization of disparities in these non-traditional risk factors can provide additional benefits in CVD outcomes in Blacks than Whites with MetS deserves further elucidation.

**Figure 1:**
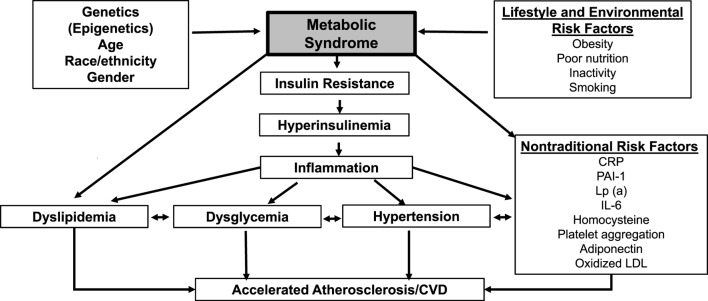
Schematic diagram of metabolic syndrome (MetS) and its contributory factors. Legend: schematic diagram of MetS and its contributory factors. C-reactive protein (CRP), plasminogen activator inhibitor-1(PAI-1), lipoprotein a [Lp (a)], interleukin 6 (IL-6), low-density lipoprotein (LDL).

In summary, there is a strong and convincing evidence to support ethnic and racial difference in MetS and its components in Blacks when compared to Whites. However, these metabolic disparities in Blacks cannot explain the excess CVD and T2DM than in Whites. These issues are particularly relevant when using traditional metabolic risk factors rather than non-traditional biomarkers (Figure 1). Thus, it is important to incorporate or include non-traditional markers, such as CRP, IL-6, adipocytokines, oxidized LDL, homocysteine, PAI-1, platelet aggregation factor, etc., in assessing the risk for CVD and T2DM in Blacks of African Ancestry who reside in diverse geographic locations (Figure 1).

## Conclusion

Racial and ethnic disparities in CVD and T2DM risk factors exist in Blacks and Whites with MetS residing in geographic locations. Although, the exact reasons are unknown, emerging evidence support genetic as well as environmental factors in the development of CVD and T2DM. Thus, further research to investigate the genetic markers and metabolic and body fat compositional parameters as well as non-traditional risk factors are needed to develop novel new definition and criteria for MetS that can predict CVD and T2DM in Blacks and Whites residing in diverse geographic locations. We believe this could provide more effective prevention and treatment of CVD and T2DM among Blacks residing in diverse geograhic locations.

## Author Contributions

Both authors have contributed to the manuscript, the literature review, analysis, interpretation and editing for the final submission.

## Conflict of Interest Statement

The authors declare that the research was conducted in the absence of any commercial or financial relationships that could be construed as a potential conflict of interest. The reviewer LB and handling editor declared their shared affiliation, and the handling editor states that the process met the standards of a fair and objective review.
